# Disruption of Hydrogen Bonds between Major Histocompatibility Complex Class II and the Peptide N-Terminus Is Not Sufficient to Form a Human Leukocyte Antigen-DM Receptive State of Major Histocompatibility Complex Class II

**DOI:** 10.1371/journal.pone.0069228

**Published:** 2013-07-25

**Authors:** Monika-Sarah E. D. Schulze, Anne-Kathrin Anders, Dhruv K. Sethi, Melissa J. Call

**Affiliations:** 1 Department of Cancer Immunology & AIDS, Dana-Farber Cancer Institute, Boston, Massachusetts, United States of America; 2 Program in Immunology, Harvard Medical School, Boston, Massachusetts, United States of America; 3 Structural Biology Division, the Walter and Eliza Hall Institute of Medical Research, the University of Melbourne, Parkville, Victoria, Australia; Johns Hopkins University, United States of America

## Abstract

Peptide presentation by MHC class II is of critical importance to the function of CD4+ T cells. HLA-DM resides in the endosomal pathway and edits the peptide repertoire of newly synthesized MHC class II molecules before they are exported to the cell surface. HLA-DM ensures MHC class II molecules bind high affinity peptides by targeting unstable MHC class II:peptide complexes for peptide exchange. Research over the past decade has implicated the peptide N-terminus in modulating the ability of HLA-DM to target a given MHC class II:peptide combination. In particular, attention has been focused on both the hydrogen bonds between MHC class II and peptide, and the occupancy of the P1 anchor pocket. We sought to solve the crystal structure of a HLA-DR1 molecule containing a truncated hemagglutinin peptide missing three N-terminal residues compared to the full-length sequence (residues 306–318) to determine the nature of the MHC class II:peptide species that binds HLA-DM. Here we present structural evidence that HLA-DR1 that is loaded with a peptide truncated to the P1 anchor residue such that it cannot make select hydrogen bonds with the peptide N-terminus, adopts the same conformation as molecules loaded with full-length peptide. HLA-DR1:peptide combinations that were unable to engage up to four key hydrogen bonds were also unable to bind HLA-DM, while those truncated to the P2 residue bound well. These results indicate that the conformational changes in MHC class II molecules that are recognized by HLA-DM occur after disengagement of the P1 anchor residue.

## Introduction

Cell surface presentation of antigenic peptides by major histocompatibility class II (MHCII) molecules to CD4+ T cells plays a pivotal role in the adaptive immune response to foreign pathogens. Antigen presenting cells not only present antigen to T cells, but also provide co-stimulatory signals that reflect the physiological context in which the antigen was acquired, and these are important indicators that determine whether tolerance should be maintained or an immune response initiated [1]. Antigen-specific T cell priming may take many hours within the lymph node [2] and during this time the peptides presented by MHCII must remain stably bound. Accordingly, MHCII:peptide complexes are extremely long-lived with half-lives of days to weeks [3,4], and empty MHCII molecules rapidly lose their ability to re-bind peptide [5,6]. While these properties are highly desirable in the surface-displayed complex, they represent significant challenges during intracellular peptide loading, when facile exchange must occur to support effective selection of the highest-affinity peptides.

The critical importance of human leukocyte antigen (HLA)-DM (DM) in the MHCII peptide-loading pathway was first determined in cells that displayed high levels of class II-associated invariant chain (CLIP) peptide on surface MHCII [7]. CLIP is a remnant of the invariant chain (a chaperone protein with which nascent MHCII molecules are folded), which acts to protect the peptide-binding groove until exchange with peptides in the endocytic pathway takes place [8,9]. Reconstitution of this mutant cell line with cDNA encoding DMβ reversed the defect, and subsequent biochemical assays determined that DM physically interacts with HLA-DR (DR): CLIP complexes to promote dissociation of the CLIP peptide [10-12]. Further studies demonstrated that DM not only acts on MHCII:CLIP complexes but can promote the exchange of any peptide [13,14].

DM can act sequentially on many MHC molecules and because of this it has been proposed that DM has enzyme-like activity [15,16]. Enzymatic reactions are typically described in terms of an initial Michaelis complex of enzyme and substrate that then results in a rearrangement of covalent bonds that elicits dissociation of a product. DM does not break or form covalent bonds in the same manner as traditional enzymes but instead disrupts non-covalent interactions that result in peptide release from the binding groove of MHCII. Mutations that alter enzymatic function can often affect the initial formation of the Michaelis complex or be localized to the active site and affect catalytic turnover. In an alternative model, DM activity may instead be described in terms of conformational selection. In this model, MHCII:peptide complexes exist in multiple conformational subspecies at equilibrium and the ratio of these conformers is determined by the properties of the bound peptide. DM has high affinity for one or more of these subspecies and by binding to a particular conformer perturbs the entire equilibrium. The remaining MHCII molecules restore the equilibrium thus creating more conformers for DM to bind. This process continues until a new equilibrium with MHCII:peptide, DM-bound MHCII and free peptide is reached. The addition of a second free peptide to the system will perturb the equilibrium in the reverse direction allowing peptide exchange to occur.

This model of conformational selection is supported by the crystal structure of the complex between DM and DR [17] (discussed in more detail below). Superposition of the structure of free MHCII onto MHCII in complex with DM shows that steric clashes would prevent free MHCII from binding DM without prior rearrangement, while DM itself shows very little rearrangement between free and bound states. Rigidity of the MHCII molecule also inversely correlates with DM susceptibility [18]. Introduction of a tyrosine into the P1 pocket by mutation of DRB1*0101 (DR1) β G86 has been shown to reduce DM binding, suggesting that DR must be able to adopt a different conformation to interact productively with DM. In surface plasmon resonance (SPR) measurements we have tested a variety of DM and DR mutants and in all cases binding affinity and catalytic activity have been directly correlated [17,19].

The role of peptide in determining MHC stability and DM susceptibility has long been appreciated [13,20-22]. The kinetic stability of peptide: MHC complexes influences DM-mediated peptide exchange, although the relationship is not strictly linear [13], implying that some interaction points between peptide and MHCII are more important than others. MHCII molecules anchor peptides through a conserved network of hydrogen bonds and a series of pockets within the peptide-binding groove [23]. The residue that engages the first pocket defines the binding register of a given peptide and is called P1. MHCII pockets typically engage the P1, P4, P6, and P9 residues while the others are solvent exposed to provide points of contact with the T-cell receptor (TCR) [23,24]. Residues N-terminal to P1 are referred to as P minus 1 (P-1), P-2 and so forth.

Hydrogen bonds at the peptide N-terminus are thought to be a deciding factor in DM susceptibility. Stratikos et al. [25], hypothesized that DM acts to break hydrogen bonds between DR and the backbone of the peptide and that artificial complexes that are biochemically unable to form these bonds would therefore be less responsive to DM. Surprisingly, complexes missing hydrogen bonds to P-2, P-1 and P1 in fact had enhanced responsiveness to DM, and thus Stratikos et al., concluded that these hydrogen bonds are likely to be broken before DM interaction rather than after. A number of studies have focused primarily on the hydrogen bond between the conserved residue DR1β H81 and the peptide backbone. Narayan et al. [26], proposed that DM binds to unstable MHC:peptide complexes and induces conformational changes that perturb the hydrogen bond supplied by DR1β H81 to eject peptide from the groove. Results from Zhou et al. [27] and Ferrante et al. [28], show that DR1β H81 is only part of the story and that DM activity is not limited to one particular hydrogen bond, but instead multiple interactions are broken to facilitate peptide release. SPR measurements [19] have indicated that the hydrogen bonds reported by Stratikos et al. [25], at the N-terminus of the peptide and dissociation of the P1 anchor is required before significant quantities of MHCII molecules adopt the conformational species recognized by DM.

The recent crystal structure of DM in complex with DR1 has provided new insights into the specific conformational changes that occur during DM-mediated peptide exchange [17]. DRα W43, which usually points toward the P1 pocket, adopts a different orientation when DM is bound and points into the DM:DR1 interface. Mutational analysis and cross-species conservation of DRα W43 and the residue it contacts, DMα N125, suggest that rotation of DRα W43 is a necessary step to DM engagement. In addition, an extended stretch of random coil situated above DRαW43 becomes helical, causing a translation of the aromatic residues DRα F51 and DR1β F89 towards the P1 pocket. These alterations can only occur after the N-terminus of the peptide has disengaged as hydrogen bonds between DRα and the peptide backbone are not compatible with the induced helical content, and DRα F51 occupies the P1 pocket. For these conformational changes to be reversed, incoming peptide must efficiently engage the P1 pocket to compete with DRα F51 and DR1β F89, regain hydrogen bonds to the peptide backbone and rotate DRα W43 back to its normal position.

The structure of the DM:DR1 complex has captured a highly informative intermediate in the peptide-loading pathway. However, we cannot deduce from this structure whether the conformation adopted by MHCII in complex with DM is formed prior to DM engagement or whether an as yet undescribed conformation of MHCII is the DM target. To determine the conformational state of a DM-responsive DR1 molecule *prior to* DM engagement, we attempted to solve the structure of DR1 with an N-terminally truncated hemagglutinin (HA) peptide that was covalently linked into the peptide-binding groove but missing the P-2, P-1 and P1 residues (pHA*^P2-P11^; see Table **1** for an explanation of peptide nomenclature). Previous SPR experiments indicated that this species had high affinity for DM [19]. Surprisingly, the DR1-peptide complex that crystallized contained an occupied P1 pocket. This species had apparently arisen during DR1 loading through preferential binding of a very minor contaminant in the synthetic peptide preparation that contained a single amino acid N-terminal extension. Close inspection of this structure revealed that, despite missing all of the hydrogen bonds to P-2 and P-1, there were no major conformational differences compared to the full-length DR1:pHA^P-2-P11^ structure [29]. This complex also bound very poorly to immobilized DM in SPR experiments. We conclude that P1 anchor disengagement in addition to poor hydrogen bond occupancy at the peptide N-terminus is required to induce a conformer of DR1 that can be recognized efficiently by DM.

**Table 1:** Peptide abbreviations.

pHA (306-318), pHA^P-2-P11^	P K Y V K Q N T L K L A T
pHA*^P2-P11^	V K Q N *C* L K L A T *K-(DNP)*
pHA*^P1-P11 [P1V]^	**V** V K Q N *C* L K L A T *K-(DNP)*
pHA*^P2-P11 [P2G]^	**G** K Q N *C* L K L A T *K-(DNP)*
pHA*^P-2-P11^	P K Y V K Q N *C* L K L A T *K-(DNP)*
pHA* ^P-2-P11 [P1V]^	P K **V** V K Q N *C* L K L A T *K-(DNP)*
pHA*^P1-P11 [P1X]^	**X** V K Q N *C* L K L A T *K-(DNP)*
pHA*^P1-P11^	**Y** V K Q N *C* L K L A T *K-(DNP)*
pHA^P-2-P11 [P5Alexa]^	P K Y V K Q **C**# T L K L A T

Peptides that are linked to DRα via a cysteine at P6 and contain a C-terminal DNP moiety are indicated with an asterisk. Superscripts denote the residue positions included in each peptide. Departures from the native pHA sequence are listed in square brackets. The P1 anchor position of each peptide is underlined. # donates the presence of maleimide linked Alexa-488.

## Results

### Crystallization of DR1 carrying a truncated HA peptide

MHCII molecules undergo conformational changes during peptide exchange [18,22,30], and the recent crystal structure of a DM:DR1 complex revealed the details of some of these conformational changes [17]. The critical piece of information that allowed this structure to be solved was the experimental evidence that DM binds strongly to DR molecules in which the P1 pocket is not engaged by peptide [19]. To determine the conformational changes that occur upon release of N-terminal peptide residues from the MHCII molecule, we examined the structure of a MHCII molecule loaded with an N-terminally truncated peptide. DR1 carrying a DRα V65C mutation was loaded with a truncated version of HA that was missing residues P-2, P-1 and P1. As removal of the P1 anchor residue greatly reduces peptide affinity for MHCII, the HA peptide also carried a cysteine substitution at P6 which allowed covalent linkage of the peptide into the peptide-binding cleft via DRα V65C. A dinitrophenol (DNP) moiety on the peptide C-terminus facilitated purification of DR1 loaded with the truncated HA-peptide (pHA*^P2-P11^). This protein crystallized readily and the structure of the DR1:pHA*^P2-P11^ was solved by molecular replacement. The 2.12 Å dataset revealed two molecules in the asymmetric unit and the structure refined to R_work_ and R_free_ values of 19.9 and 23.7% respectively (see Table 2).

**Table 2:** Data collection and refinement statistics.

**Data collection**	
Space group	C222_**1**_
Resolution (Å)	50.0 - 2.12 (2.16 - 2.12)^a^
**Unit cell dimensions**	
a, b, c (Å)	95.81, 111.38, 211.54
α, β, γ (^°^)	90, 90, 90
Wavelength (Å)	1.0332
No. molecules in asymmetric unit	2
Completeness (%)	94.5 (88.9)
Multiplicity	6.1 (5.9)
R_merge_	0.056 (0.311)
<I/σI)>	28.7 (5.1)
**Refinement**	
Resolution range (Å)	49.3 - 2.12
No. reflections	58717
*R*_work/_ *R*_free_	0.199/0.237
No. protein atoms	6153
No. peptide atoms	175
No. of waters	190
**Average B-factor (Å^2^)**	
Protein	26.0
Peptide	33.2
Water	27.0
**Geometry**	
R.m.s. deviation bonds (Å)	0.008
R.m.s. deviation angles (^°^)	1.144
Ramachandran plot (%)^b^	98.8, 1.2, 0.0

a Values in parentheses are for the highest resolution shell.

^b^ Favored, allowed and disallowed regions of the Ramachandran plot [44].

**Figure 1:**
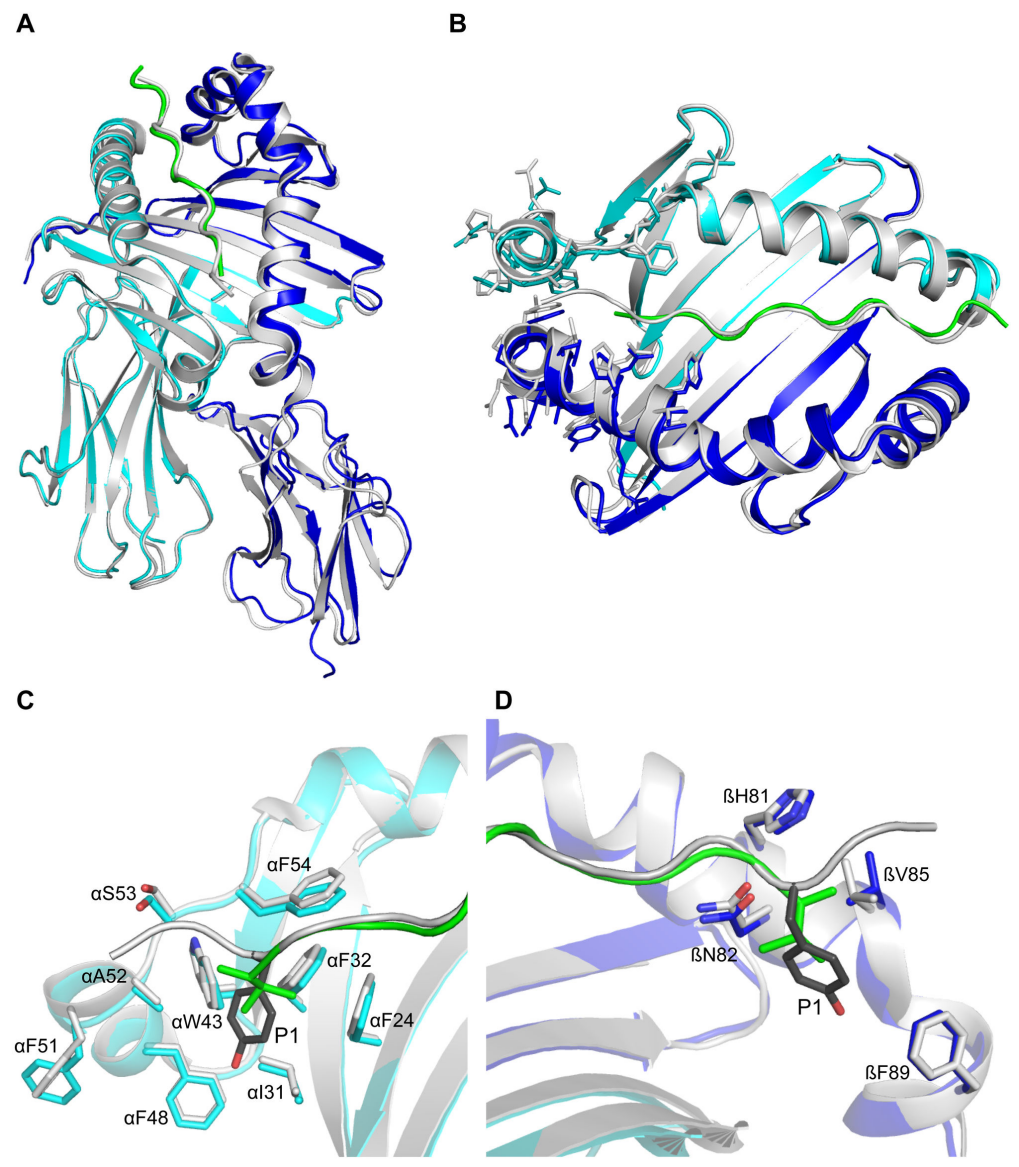
Structural comparison of DR1 carrying truncated and full-length peptide. (A–D) Overlay of DR1 carrying a truncated HA peptide variant (pHA*^P1-P11 [P1V]^) missing N-terminal residues P-2 and P-1 (DRα cyan, DR1β blue, peptide green) and DR1 carrying full-length HA peptide (pHA^P-2-P11^, gray, 1DLH). (A) Side view of overlaid DR1 ectodomains. (B) Top view of overlaid peptide-binding grooves with side chains near the peptide N-terminus shown in stick representation. (C) Close-up view of selected DRα chain residues shown in stick model near the P1 anchor residue. (D) Close-up view of selected DR1β chain residues shown in stick model near the P1 anchor.

We overlaid this structure with other DR1 structures within the protein database and saw little difference in backbone or side chain conformations of any residues. We paid close attention to DRα W43, which undergoes a major rotation in the crystal structure of DM:DR1, and residues on DRα and DR1β that flank and support the peptide N-terminus. No significant variations were seen either in terms of conformation or B-factors (see Figure 1).

### The P1 pocket is occupied in the crystal structure

#### A peptide contaminant is preferentially bound by DR1

**Figure 2:**
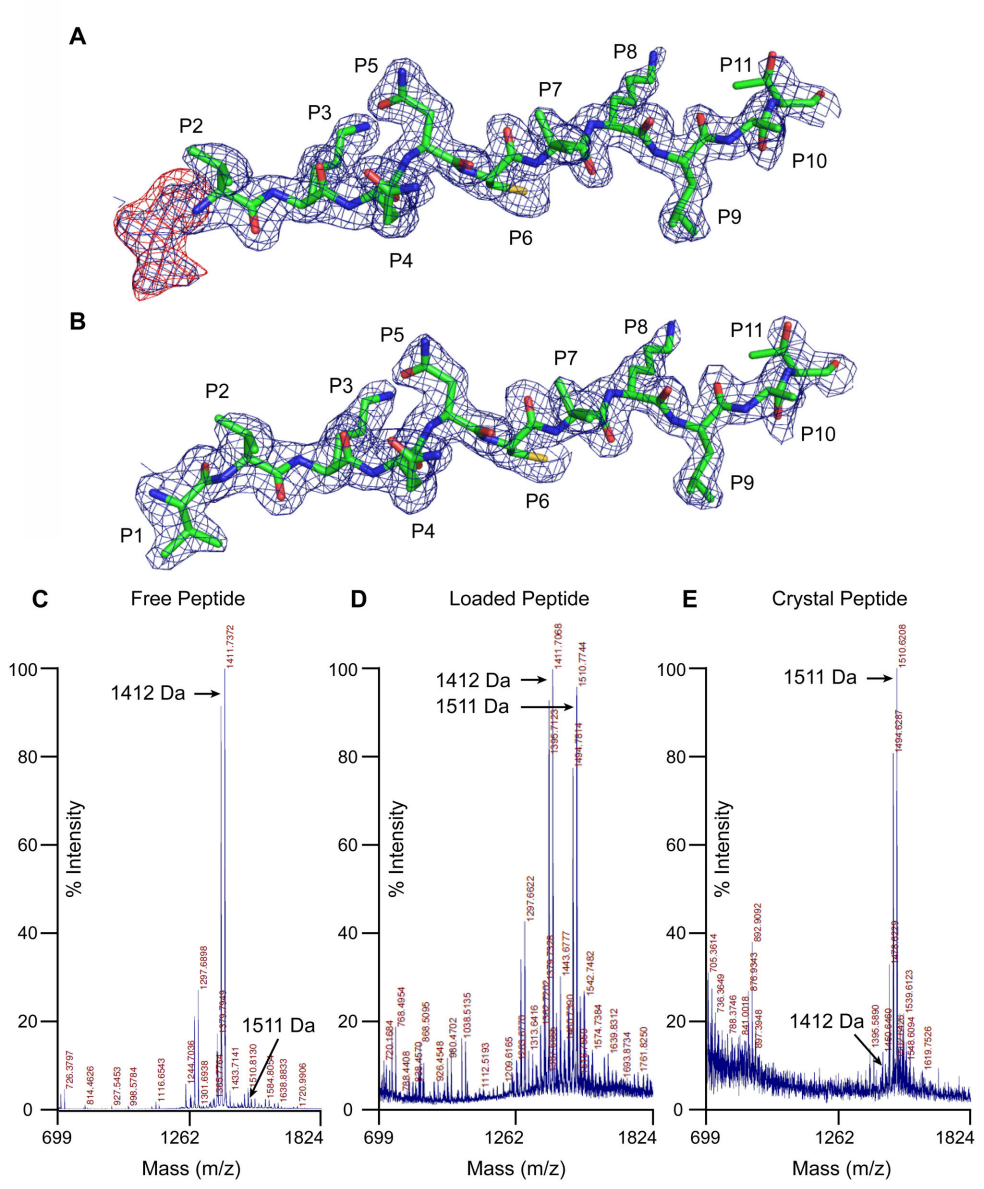
Peptide side-product with additional valine at P1 position binds in groove. (A, B) Observed 2F_o_-F_c_ and F_o_-F_c_ electron density maps contoured to 1σ and 3σ, respectively, when calculated for; (A) model containing HA variant peptide missing P-2, P-1 and P1 residues, (B) model of contaminating peptide with additional valine at P1. Peptide residue positions are indicated. (C, D, E) Mass spectra of synthesized peptide pHA*^P2-P11^; prior to DR1 binding (C), eluted from DR1 after peptide loading (D) and after crystallization (E). Masses for peptides missing the P1 residue (VKQNCLKLATK-DNP, 1411.7 Da) and containing an additional valine at P1 position (VVKQNCLKLATK-DNP, 1510.8 Da) are indicated.

Unexpectedly, when the electron density corresponding to the peptide was examined, significant density in the area of the P1 pocket was observed (see Figure 2A). The extra density was continuous with the predicted N-terminus of the peptide and thus was unlikely to be a solvent component of the mother liquor. We therefore sought to identify whether the peptide was covalently modified. Matrix-assisted laser desorption/ionization – time of flight (MALDI-TOF) analysis of the peptide used to load DR1 molecules showed a major peak at the correct theoretical molecular weight and was consistent with the expected purity of the peptide purchased (at least 80%; see Figure 2C). We again loaded DR1 molecules with pHA*^P2-P11^ and purified the complexes by size exclusion, anti-DNP affinity and anion exchange chromatography. Peptides were then eluted in the presence of dithiothreitol, purified and analyzed by MALDI-TOF. This revealed two peaks of roughly the same intensity; one corresponding to the expected molecular weight of pHA*^P2-P11^ (1412 Da) and one that was 99 Daltons larger (1511 Da) (see Figure 2D). Closer inspection of the spectrum of free peptide revealed that the 1511 Da peak was present, but had been greatly enriched during the peptide loading reaction. We therefore concluded that our synthesized peptide contained a minor peptide contaminant that had a competitive advantage in the loading reaction compared to the desired peptide. The difference in mass between the two peaks corresponded precisely to one valine residue and a peptide containing an extra valine at the N-terminus fitted the calculated density well. Peptide sequencing of the 1511 Da peak by liquid chromatography-tandem mass spectrometry also indicated an extra valine at the N-terminus. Analysis of the protein used for crystallization suggested that approximately half of the DR1 molecules contained the expected peptide, while the other half had bound the contaminant, assuming the two peptides behaved similarly in the MALDI-TOF analysis.

#### DR1 containing the longer peptide is the complex that crystallizes

If DR1 contained a mixture of the two peptides and both species were incorporated into the crystal, then the atoms at the P1 pocket should have 50% occupancy. To determine whether the electron density observed was weaker than expected, we set the occupancy of the P1 valine residue at 0.5 and performed three rounds of occupancy refinement in Phenix [31]. In each case the occupancy reverted back to a value close to 1. We then isolated crystals of DR1:pHA*^P2-P11^ and analyzed the peptide present by MALDI-TOF and found that the molecular weight of the peptide was almost exclusively 1511 Da, with the peak of the desired peptide barely detectable (see Figure 2E). These results indicated that the longer peptide was enriched during peptide exchange and subsequently enriched further during crystallization. We ordered a new peptide preparation at high purity containing a glycine at P2 (pHA*^P2-P11 [P2G]^) and formed new DR1:peptide complexes for crystallography. In contrast to the original protein preparation, we were unable to obtain crystals from this new protein sample despite extensive screening.

#### DRα C-terminus contacts the peptide-binding site of a neighboring molecule

**Figure 3:**
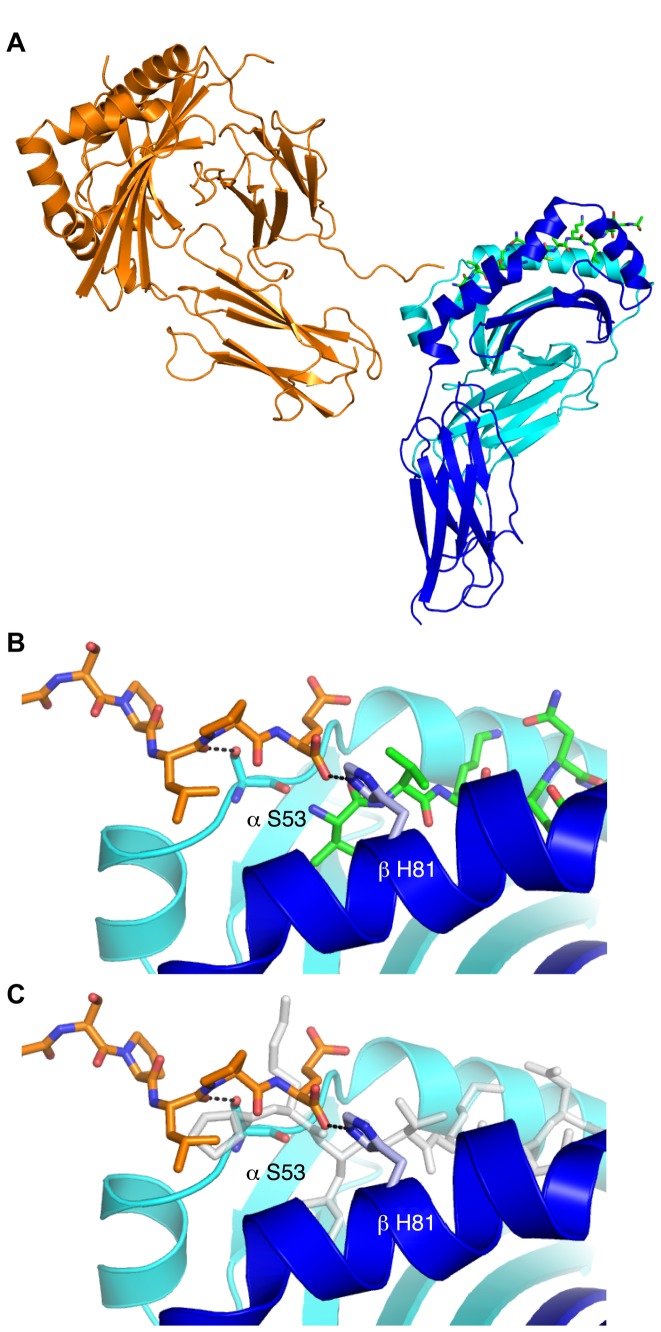
Flexible C-terminus of DR1 molecule binds in partially empty peptide-binding groove of adjacent DR1 molecule. (A) Two molecules in the crystal lattice are shown depicting a contact formed by DRα (orange) C-terminus of one DR1 molecule binding to the partially empty peptide-binding groove of the other DR1 molecule (cyan/blue). (B) Close-up view of the interactions between the neighboring DRα C-terminus (orange) and the DR1 peptide-binding groove (DRα cyan, DR1β blue, peptide green). Hydrogen bonds are displayed as dotted lines. (C) Similar view as (B), but overlaid with DR1 containing full-length HA peptide (gray). The DRα C-terminus occupies an overlapping space as P-2 and P-1 of the full-length peptide.

DR1 typically crystallizes as a “dimer of dimers” and the crystal structure presented here is no exception. However, one contact between molecules in the crystal is of particular interest. As illustrated in Figure **3A& B**, the flexible C-terminus of the DRα chain in one molecule binds to the partially empty peptide-binding groove of an adjacent DR1 molecule. When the structure of DR1 with full-length HA peptide (pHA^P-2-P11^) is overlaid with this molecule in the asymmetric unit, it is apparent that the C-terminus of the neighboring molecule occupies similar space as the pHA^P-2-P11^ N-terminus (see Figure 3C). This distinctive intermolecular contact between adjacent DR1 molecules occupies only one of the two peptide-binding grooves in the asymmetric unit and is stabilized by similar hydrogen bonds as the pHA^P-2-P11^ N-terminus. DR1β H81, which usually interacts with the peptide backbone at residue P-1, forms a hydrogen bond to the neighboring DRα C-terminal carboxyl group. Furthermore, the side chain of DRα S53 forms a hydrogen bond to the backbone of the third-to-last residue of the DRα C-terminus (DRα L211). Root mean square deviation analysis between each DR1 molecule in the asymmetric unit reveals no substantial deviations in structure between the two molecules (0.4623 Å Cαatoms 1.0032 Å all atoms), despite these differences in crystal contacts.

### Comparison of the N-terminal hydrogen bond network with the full-length DR1:HA structure

While we were unable to solve a crystal structure of DR1 without a P1 anchor, we did obtain a crystal structure where key hydrogen bonds between MHC and the peptide backbone that have previously been implicated in DM activity [25] were absent due to the truncation of P-2 and P-1. Removal of hydrogen bonds from the DRα F51 and DRα S53 main-chain carbonyls to these residues increased the susceptibility of DR1 to DM-mediated peptide exchange by 6.2-fold [25], but it is unclear whether this effect was primarily due to disruption of these hydrogen bonds or to a reduction in global stability at the peptide N-terminus.

**Figure 4:**
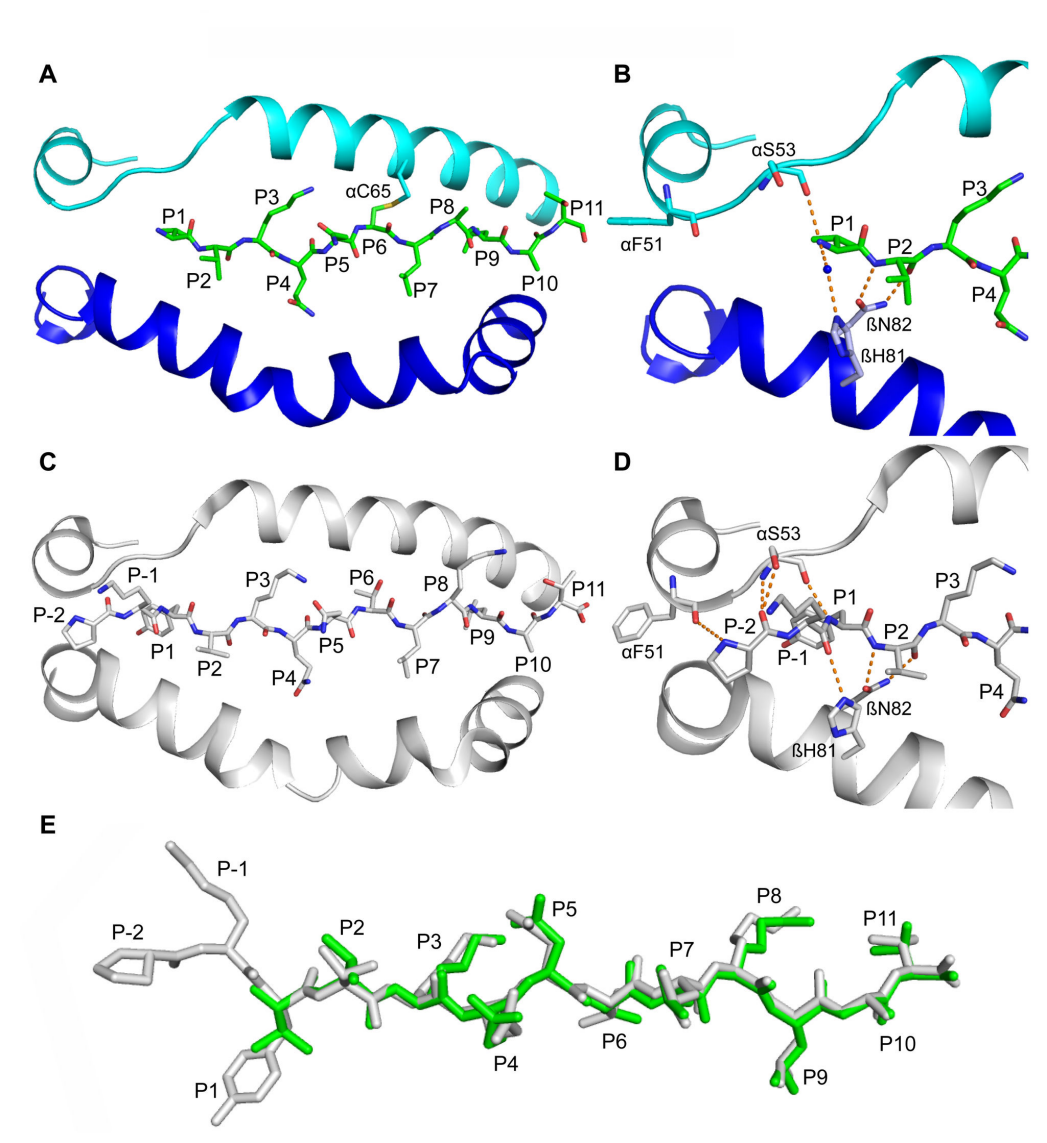
Absence of two N-terminal peptide residues (P-2, P-1) causes no major conformational change of peptide-binding groove. (A) Top view of peptide-binding groove with pHA*^P1-P11 [P1V]^ linked at αC65 (DRα, ribbon, cyan; DR1β, ribbon, blue; peptide, stick, green). (B) Close-up view of the peptide N-terminus of the structure shown in (A). Hydrogen bonds between DR1 residues and the peptide N-terminus are shown with dotted lines. A water molecule coordinating a hydrogen bond from DR1β H81 to the peptide N-terminus is shown as a blue sphere. (C) Top view of peptide-binding groove with full-length pHA^P-2-P11^ (ribbon and stick, gray; 1DLH). (D) Close-up view of the peptide N-terminus of the structure shown in (C). Hydrogen bonds between DR1 residues and the peptide N-terminus are shown with dotted lines. (E) Superimposition of truncated (green) and full-length (gray) peptide.

In the full-length DR1:pHA^P-2-P11^ structure, the carbonyl of DRα F51 forms a hydrogen bond with the amide nitrogen of P-2, while the amide nitrogen and side chain hydroxyl of DRα S53 hydrogen bonds to the carbonyl of P-2 and the DR1β H81 imidazole hydrogen bonds to the P-1 carbonyl [29] (see Figure 4D). Due to the peptide truncation, these hydrogen bonds are absent in the structure presented here (DR1:pHA*^P1-P11 [P1V]^) and DR1β H81 instead forms a hydrogen bond to a water molecule that bridges DR1β H81 and the peptide N-terminus. The other three conserved N-terminal hydrogen bonds in this area, formed by peptide residues P1 and P2 with DR1 residues DRα S53 and DR1β N82, respectively, are unmodified (compare Figure 4B and 4D). MHCII residues flanking and forming the P1 pocket exhibit no change in position (see Figure 1). Comparison of full-length DR1:pHA^P-2-P11^ and this structure (DR1:pHA*^P1-P11 [P1V]^) did not reveal increases in B-factors in the regions implicated in conformational changes during DM interaction.

### Breaking the hydrogen bond network extending from DRα F51 to S53 is not sufficient for DM binding in the presence of the P1 side chain

**Figure 5:**
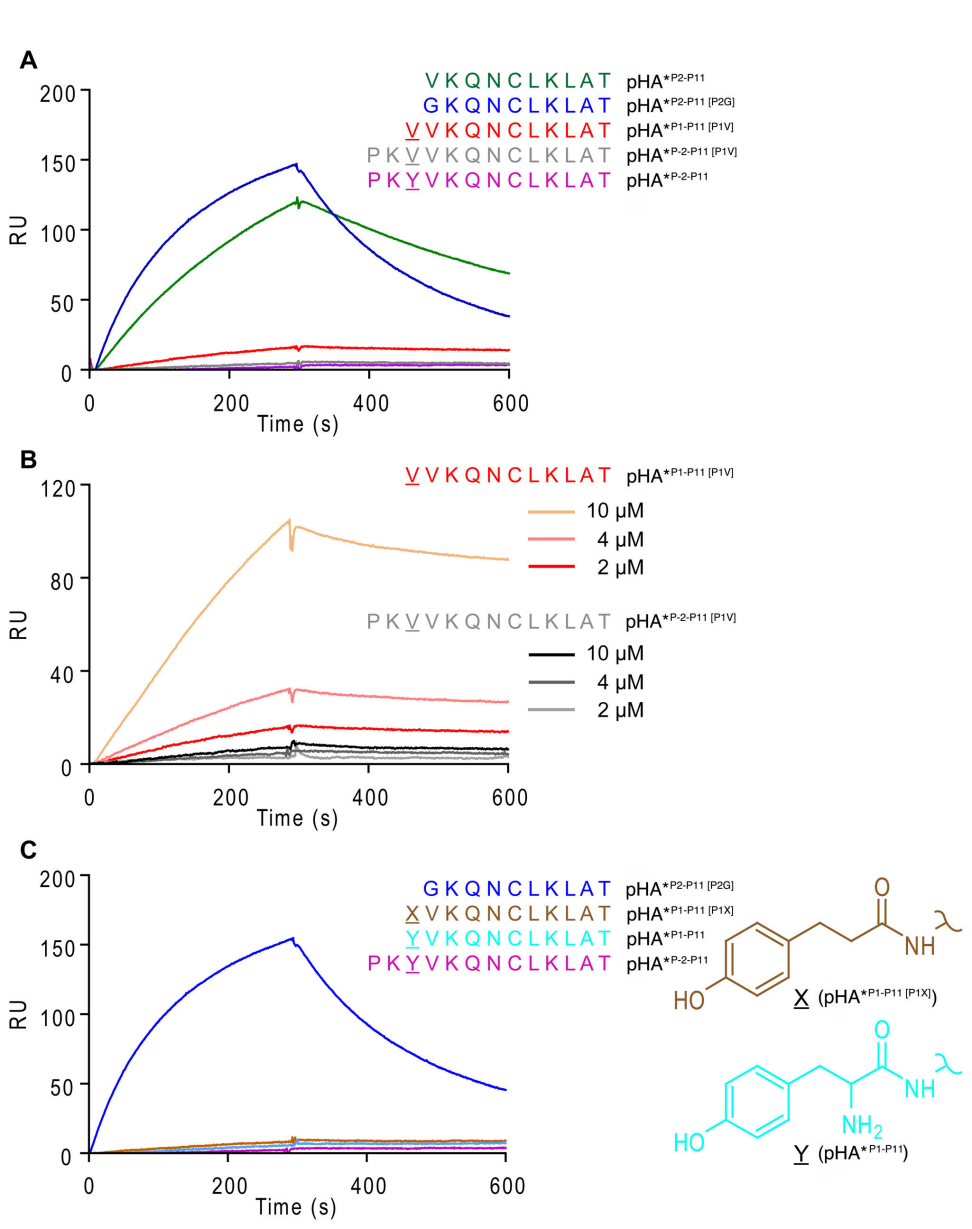
Disrupting interactions between P1 side chain and P1 pocket is crucial for DM binding. (A–C) SPR binding curves for DR1 molecules covalently linked to the P6 position of peptides indicated at the right of each graph. Each curve and peptide have the same color. DR1/peptide complexes (2 µM or indicated concentration) were injected for 5 minutes (pH 5.3, 15 µL/min, 25 ^°^C), followed by buffer injection for an additional 5 minutes. Overall binding (RU) was calculated by subtracting the background binding to a flow cell containing DM mutant (R98A, R194A), which is unable to interact with DR from the specific binding from the flow cell containing wild-type DM.

As DR molecules missing hydrogen bonds at the peptide N-terminus are more susceptible to DM [25], we would assume that the species that crystallized has higher affinity for DM than DR1 containing full-length peptide, but we could find no evidence of conformational changes that might be recognized by DM. To further delineate the contribution of hydrogen bonds to DM susceptibility, we tested different DR1:peptide complexes (all covalently stabilized through a cysteine at P6) for their ability to bind to DM by SPR. First, we loaded two HA variants that were missing P1 anchors. The first had a valine at P2 and was identical to the peptide preparation used for our crystallization trials (pHA*^P2-P11^). This complex exhibited measurable binding to DM at 2 µM (Figure **5A**, green trace). From our previous analysis of peptide loading using this preparation of pHA*^P2-P11^ (Figure 2D), we predicted that the resulting complexes would contain a mixture of peptides with the desired product (pHA*^P2-P11^) and the N-terminally extended contaminant (pHA*^P1-P11 [P1V]^) in approximately equal amounts. To determine the contribution of the contaminating species to the binding curves, we loaded DR1 with a pure preparation of

pHA*^P1-P11 [P1V]^. Mass spectrometry analysis confirmed only the expected peptide was crosslinked into the binding site (data not shown). DR1:pHA*^P1-P11 [P1V]^ exhibited almost no binding to DM when injected at 2 µM (Figure **5A**, red trace), indicating that the binding observed using pHA*^P2-P11^ (Figure **5A**, green trace) was contributed almost entirely by DR1 containing peptide without a P1 anchor. We then tested complexes loaded with peptide containing glycine at P2 (pHA*^P2-P11 [P2G]^). This complex exhibited strong binding (Figure **5A**, blue trace), again confirming that DR1 complexes containing an unoccupied P1 pocket are preferred binding partners for DM and that truncation of P-2 and P-1 is not sufficient to induce strong binding. Control DR1 complexes loaded with full-length pHA had no detectable binding to DM when either valine (pHA*^P-2-P11 [P1V]^, Figure **5A**, gray trace) or tyrosine (pHA*^P-2-P11^, Figure **5A**, magenta trace) occupied the P1 position.

We further investigated the role of residues at the P-2 and P-1 positions by directly comparing different concentrations of DR1 loaded with either pHA*^P1-P11 [P1V]^ or pHA*^P-2-P11 [P1V]^, both of which contained valine at position P1 (Figure 5B). While dose-dependent binding could be measured between DM and DR1 molecules containing pHA*^P1-P11 [P1V]^ (Figure 5B, salmon, pink and red traces), no binding was observed when P-2 and P-1 residues were present (Figure 5B, grayscale traces). Only at 5-fold higher concentrations could binding of DR1:pHA*^P1-P11 [P1V]^ approach the binding capacity of DR1:pHA*^P2-P11^.

The crystal structure of DR1 presented here indicates that the amide nitrogen of the P1 anchor makes a hydrogen bond to the carbonyl oxygen of DRα S53 and that DR1β H81 can coordinate a water molecule which hydrogen bonds to the peptide N-terminus. To disrupt these hydrogen bonds in addition to those involving P-2 and P-1 without vacating the P1 anchor position, we synthesized a peptide with a non-natural amino acid at the N-terminus that resembles tyrosine but is missing the N-terminal amine group (pHA*^P1-P11 [P1X]^, See Figures **5C** and 4B). When this species was loaded into DR1 molecules, binding to DM was still undetectable by SPR (Figure 5C, brown trace). So while hydrogen bonds to the peptide N-terminus influence the interaction between DR and DM, these data suggest that the interaction is significantly more sensitive to the occupancy of the P1 pocket.

### Destabilization of the peptide N-terminus is critical for DM activity

**Figure 6:**
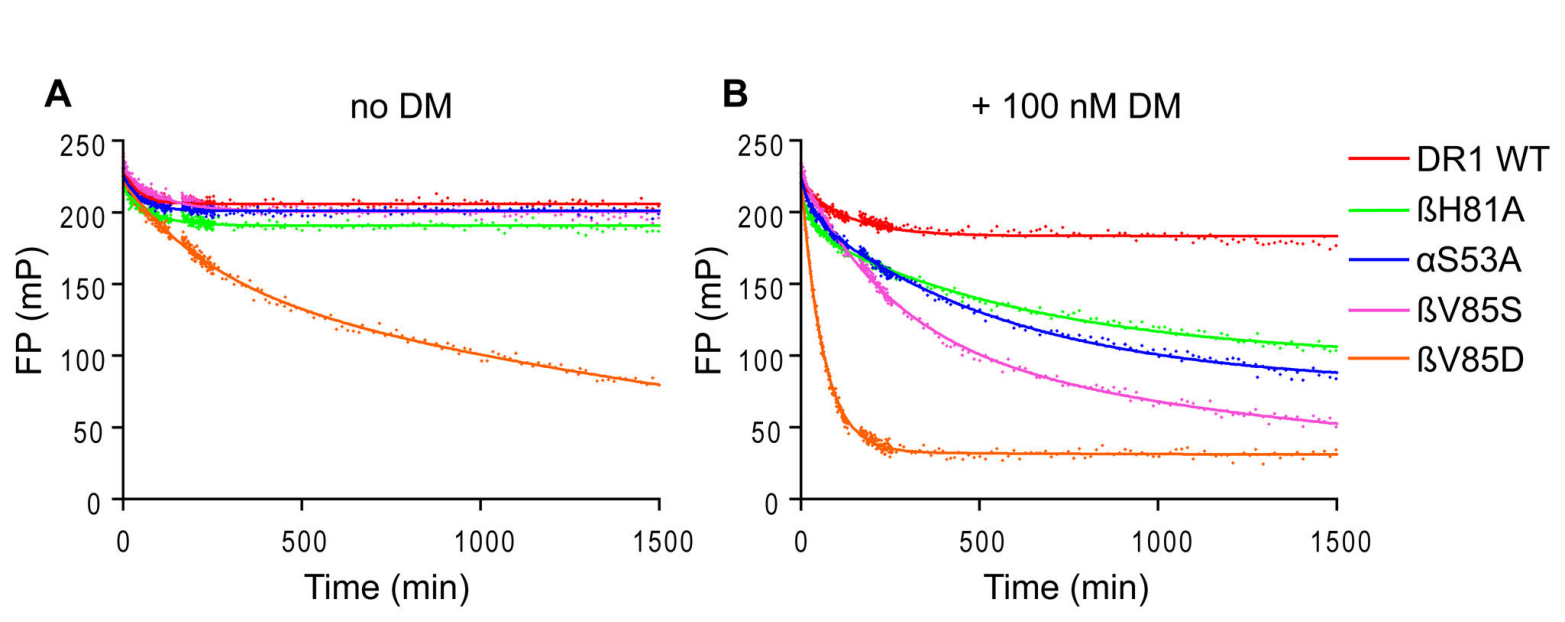
DM-catalyzed peptide release is enhanced by mutations that weaken interactions between the peptide N-terminus and DR1. (A, B) DR1β mutants H81A, V85S and V85D and DRα mutant S53A were compared with wild type DR1 complexes in peptide dissociation assays. Dissociation at pH 5.3 and 25 ^o^C of Alexa-488-labeled full-length pHA^P-2-P11 [P5Alexa]^ peptide from preloaded DR1 complexes (100 nM) in the presence of 50 µM unlabeled pHA^P-2-P11^ competitor peptide was measured by FP. Dissociation curves were preformed in the absence (A) or presence (B) of 100 nM DM. FP values are presented in millipolarization units (mP).

We hypothesized that individual hydrogen bonds at the peptide N-terminus were less important than global stability of the peptide N-terminus, which secures the P1 anchor residue in place. DR1 loaded with full-length pHA^P-2-P11^ is extremely stable and highly resistant to DM-mediated peptide exchange. We tested several DR1 mutants that were designed to destabilize the peptide N-terminus in different ways in an established fluorescence polarization (FP) assay that measures peptide release [32] in the presence or absence of DM. DR1 was loaded with full-length Alexa-488-labeled HA peptide (pHA^P-2-P11 [P5Alexa]^) and dissociation was measured in the presence of excess unlabeled pHA^P-2-P11^ by FP (see Figure 6). As expected, unmodified DR1 (Figure 6, red trace) showed very little peptide exchange in either the absence (Figure 6A) or presence (Figure 6B) of 100 nM DM over the time course of the experiment (~24 hours). DRα S53 and DR1β H81 were chosen for mutation to alanine as both are able to form hydrogen bonds via their side-chains to the peptide backbone. Both mutants retained peptide during the 24-hour time-course similarly to wild-type DR1 but were more responsive to DM-mediated peptide release (Figure 6, green and blue traces). DR1β V85 points towards the P1 pocket and is surface exposed in our crystal structure. In the previously solved structure of DR1 with full-length HA peptide [29], DR1β V85 is shielded from solvent by the HA P-2 residue. We therefore altered the hydrophilicity and electrochemistry of the P1 pocket and surrounding area by making separate mutations to serine (Figure 6, pink traces) and aspartic acid (Figure 6, orange traces). DR1β V85S was well tolerated and displayed robust binding to HA peptide in the absence of DM. When DM-mediated peptide dissociation was measured, however, this mutant responded more strongly to DM than WT DR1. DR1β V85D displayed a reduced affinity for peptide, showing significant peptide loss over 24 hours even in the absence of DM. This mutant was still sensitive to catalyzed peptide exchange as addition of DM clearly accelerated peptide dissociation. Thus, in each case tested here, mutants predicted to destabilize the peptide N-terminus, whether through removal of hydrogen bonds or deformation of the P1 pocket, resulted in enhanced susceptibility to DM-mediated peptide exchange.

## Discussion

It is clear from a number of lines of evidence that the peptide N-terminus is important for whether DM targets DR:peptide complexes for peptide exchange or not [19,25-27]. Peptides are stabilized along their entire length by a conserved network of hydrogen bonds and anchored at strategic points by pockets within the peptide-binding groove [23], but analyses of these interactions have shown that much of the binding energy holding a peptide in the groove is mediated by hydrogen bonds at the peptide N-terminus [33,34] and the P1 anchor residue [35]. It is perhaps not surprising, then, that the lateral face of DR with which DM interacts is centered towards the peptide N-terminus [17,36-38].

Biochemical studies of DM function have demonstrated that DM promotes the dissociation of peptide, chaperones empty molecules and edits the peptide repertoire [10-12,39-41]. The recent crystal structure of DM in complex with DR1 provides a mechanistic explanation to these functions. Structural rearrangements in the peptide-binding groove of DR1 in this co-crystal structure result in DR1’s own residues, specifically DRα F51 and DR1β F89, occupying the P1 pocket [17]. These changes hold the groove open and allow free peptides to sample the groove, and only those that can compete out DRα F51 and DR1β F89 will bind securely.

What is missing from our knowledge of this critical step in the peptide-loading pathway of MHC class II is a structural understanding of the species that DM binds. Binding studies have indicated that truncation of the peptide N-terminus up to and including the P1 anchor residue is required to measure a robust interaction between DM and DR [19]. Biochemical studies that measure peptide exchange suggest that even modest changes that interfere with the hydrogen bond network at the peptide N-terminus are sufficient to sensitize a DR:peptide complex for DM action [25]. We have solved a crystal structure of DR1 crosslinked to a truncated peptide that is incapable of forming hydrogen bonds that have been implicated in DM sensitization. In this structure, we see no evidence of conformational changes that might promote an interaction with DM. Therefore, we must look to an alternative explanation as to why DM is able to act more efficiently on MHCII:peptide complexes missing select hydrogen bonds to the peptide N-terminus than those with more stable peptide-binding properties.

To further address this conundrum we turned to SPR to directly measure the affinity of various DR1:peptide complexes to DM. While crystallography provides a static snapshot of the most abundant and stable conformation of DR1:peptide in solution, SPR will only report on species that can bind to DM and are in significant enough quantities to elicit a signal. When we measured direct DM binding of the species that crystallized, DR1:pHA*^P1-P11 [P1V]^, we found a 5-fold reduction in binding compared to our index complex DR1:pHA*^P2-P11^. Comparison of DR1:pHA*^P1-P11 [P1V]^ to other structures that are unable to bind to DM in the SPR assay (specifically DR1:pHA^P-2-P11^) revealed no conformational changes to explain the difference in DM affinity. The most likely explanation for this binding is that only a small portion of DR1:pHA*^P1-P11 [P1V]^ is in a DM-responsive conformation and this conformation is not represented in the crystal structure. As DR1:pHA*^P2-P11 [P2G]^ had much greater binding than DR1:pHA*^P1-P11[P1V]^, removal of the P1 anchor residue results in a higher percentage of the DR1:peptide complex adopting a DM-responsive conformation.

Proteins are in constant motion and the peptide bound within the groove is no exception. Even the most stably bound peptide will sample states of partial binding, although much less often than peptides with poor stability. Removal of peptide-stabilizing N-terminal hydrogen bonds will lead to an increase in sampling of partial binding states (e.g., detachment of the peptide N-terminus) and provides an explanation for the observation that removal of N-terminal hydrogen bonds are sufficient to sensitize complexes for DM action. To investigate whether this might be the case, we used a peptide dissociation assay to determine if any destabilizing changes at the peptide N-terminus would increase susceptibility to DM. While crystallography and SPR primarily measure the most abundant conformers in solution, this irreversible kinetic assay (unlabeled peptide is in excess) performed over long time periods is sensitive to even tiny populations of DM-sensitive DR1 conformers, provided conformational exchange between states is possible during the time course of the experiment. In these experiments we did not covalently link peptide in the binding groove, but instead measured the effect of DR1 mutations near the peptide N-terminus. We mutated DRα S53 and DR1β H81, which each contribute a hydrogen bond to the peptide N-terminus from their respective side chains and changed the electrostatics of the P1 pocket by mutating the buried DR1β V85 residue. In each case DM had a greater activity on destabilized complexes compared with the index complex DR1:pHA^P-2-P11^. Furthermore, if movement of the peptide N-terminus is restricted by introduction of a tyrosine at P1, which has greater complementarity to the P1 pocket than valine, we would expect that DM binding would be reduced, and this was also found to be the case. In addition, other methods to stabilize the peptide N-terminus including inclusion of P-1 and P-2 residues, which can engage conserved hydrogen bonds to the DR1 residues lining the peptide-binding site also reduced DM binding. Thus we conclude that disengagement of hydrogen bonds at the peptide N-terminus is not *sufficient* to induce bulk conformational changes that allow DM binding, but rather act to increase the likelihood of P1 anchor disengagement.

It is typically difficult to crystallize proteins that are conformationally heterogeneous, however conformational heterogeneity can sometimes be captured. Painter et al, recently reported the crystal structure of DR1 carrying a mutation in DRα F54 near the P1 pocket [34]. The DM:DR structure [17] shows that this residue undergoes a significant conformational change during peptide exchange and rotates up and out of the P1 pocket to become solvent exposed. One of the molecules in the asymmetric unit of the DRα F54C structure, displays an altered conformation of residues near the peptide N-terminus, reminiscent of the more substantial conformational changes seen in the DM:DR structure. The structure of DRα F54C clearly captures one of the conformational intermediates that MHC molecules constantly sample, although DRα W43 remained in place, indicating further conformational changes are required to bind DM.

The crystal structure of DR1 containing peptide without a P1 anchor remains an important and challenging structural target for understanding DM-mediated peptide exchange, but successful crystallization will require a strategy that addresses a number of hurdles. We found that the P1 pocket has such a strong preference to be filled that contaminating peptide sequences with poor P1 anchors will be selectively bound if available during the loading reaction. In addition, flexible polypeptide stretches in the DR1 molecules themselves may occupy the peptide-binding groove during crystallization, as observed in one (but not both) of the molecules of the asymmetric unit in the structure presented here. Selective crystallization of the species of DR1 carrying a peptide with a P1 anchor may indicate that partially empty molecules have increased structurally heterogeneity. While the step-wise conformational changes of peptide exchange remain to be fully elucidated, the work presented here indicates that breaking the hydrogen bond network at the peptide N-terminus does not in itself elicit a conformational change in the bulk of DR1:peptide complexes in solution.

## Materials and Methods

### Peptides

The peptide (pHA*^P2-P11^; see Table 1) used for crystallization studies had the sequence VKQNCLKLATK(DNP), where K(DNP) is a lysine residue derivatized with DNP and was provided at a purity of 80.13% (Peptide 2.0, Chantilly, VA, USA). The following peptides used in SPR experiments (which have the P1 anchor underlined) were purchased from 21^st^ Century Biochemicals, Marlboro, MA, USA and had these sequences (purity): pHA*^P2-P11 [P2G]^, GKQNCLKLATK(DNP) (94.25%), pHA*^P1-P11 [P1V]^, VVKQNCLKLATK(DNP) (75.70%), pHA^*P-2-P11 [P1V]^, PKVVKQNCLKLATK(DNP) (74.83%), pHA*^P1-P11 [P1X]^, XVKQNCLKLATK(DNP) (75.15%, where X is 4-hydroxyphenylpropionic acid), The remaining peptides were purchased from Peptide 2.0, Chantilly, VA, USA. pHA*^P-2-11^, PKYVKQNCLKLATK(DNP) (86.68%), pHA*^P1-P11^, YVKQNCLKLATK(DNP) (86.55%). FP experiments utilized pHA^P-2-P11[P5Alexa]^ PKYVKQC(Alexa-488) TLKLAT, where C(Alexa-488) is a cysteine residue conjugated to Alexa-488 via a maleimide linkage. This peptide and its unlabeled counterpart pHA^P-2-P11^ PKYVKQNTLKLAT were also ordered from Peptide 2.0 (Chantilly, VA, USA).

### DR1 production and purification

The ectodomain of DR1 was expressed in baculovirus infected Sf9 ( *Spodoptera* *frugiperda* ) insect cells. FOS and JUN were fused to the respective DRα and DR1β C-termini to facilitate folding during expression. Low-affinity CLIP peptide (CLIP_low_, SKARMATG ALAQA; substituted anchor residues are underlined) was fused to the DR1β N-terminus via a 13 amino-acid flexible linker containing an endoproteinase GluC protease cleavage site. Mutants were created by site-directed mutagenesis using a QuikChange Lightning site-directed mutagenesis kit (Agilent Technologies). Protein was harvested from Sf9 culture media 3 days after infection with baculovirus and was purified by affinity chromatography with a monoclonal antibody to DR (L243; American Type Culture Collection). Protein was eluted from L243 conjugated sepharose using 50 mM CAPS pH 11.5 and neutralized with the addition of sodium phosphate buffer pH 6.0. Aggregated material was removed by gel filtration (Superose 6, GE Healthcare). The covalently linked CLIP_low_ peptide and C-terminal leucine zippers were cleaved with GluC containing a His-tag (New England BioLabs Inc.). The protease was removed with Ni^2+^-NTA beads (Sigma-Aldrich) and protein was further purified by anion exchange chromatography (MonoQ, GE Healthcare). Using the small molecule J10 [32] to promote peptide exchange, cleaved CLIP_low_ peptide was exchanged for HA peptide variants labeled with DNP via a lysine side chain at the peptide C-terminus and containing a P6 cysteine (21^st^ Century Biochemicals, Marlboro, MA, USA; Peptide 2.0, Chantilly, VA, USA). Covalent attachment of the peptide to the cysteine introduced at position 65 of DRα chain, was performed at pH 8.0 in redox conditions using a mixture of oxidized and reduced glutathione as described previously [19]. The complex was further purified by gel filtration to remove free peptide, anti-DNP affinity chromatography to isolate complexes with the loaded peptide and anion exchange chromatography (MonoQ, GE Healthcare).

DM was also expressed in baculovirus infected Sf9 cells as previously described [19]. Briefly, soluble DM proteins carrying a C-terminal BirA tag (GLNDIFEAQKIEWHE) on DMα and a C-terminal protein C tag (EDQVDPRLIDGK) on DMβ were expressed in Sf9 insect cells and purified by anti-Protein C affinity chromatography (Roche Applied Science). DM was bound to anti-Protein C agarose in the presence of 2 mM Ca^2+^ and eluted in Tris buffered saline containing 5 mM EDTA. DM was enzymatically biotinylated at the BirA tag using biotin ligase and finally purified by gel filtration (Superose 6, GE Healthcare).

### Crystallization, Data Collection and Structure Determination

For crystallization, purified DR1:pHA*^P2-P11^ was dialyzed against 10 mM MES buffer (pH 6) and concentrated to ~10 mg/mL. Initial screening plates were set up at the Collaborative Crystallization Center in Melbourne, Australia, using the nano-dispensing crystallization robots Mosquito Crystal (TTP LabTech) and Phoenix (Rigaku). Final crystals of DR1:pHA*^P2-P11^ were grown in 100 mM sodium acetate (pH 4.3), 9% PEG 10,000 at 20 ^°^C using hanging drop vapor diffusion. Crystals were mounted using MicroLoops E (Mitegen) and transferred into precipitant solution supplemented with 38% (v/v) ethylene glycol and flash frozen in liquid nitrogen. X-ray diffraction data were collected at 100 K at beamline 23-ID–D of the General Medicine and Cancer Institutes Collaborative Access Team (GM/CA-CAT) at the Advanced Photon Source (Argonne National Laboratory, IL, USA) performing a continuous vector scan over the crystal. Crystals diffracted up to 2.12 Å and diffraction data were collected from a single crystal at a wavelength of 1.0332 Å using a CCD detector (MARmosaic 300). Data were indexed and scaled using the software HKL2000 [42]. Statistics are reported in Table 2. The structure of DR1 with the truncated HA peptide was solved by molecular replacement with Phaser [43] using the previously solved DR1:pHA^P-2-P11^ structure (PDB entry code 1DLH) [29] as the search model. The structure was refined using Phenix [31] and evaluation of the structure was carried out with MolProbity [44]. During refinement manual adjustments of the structure were performed using the software Coot [45]. To assess the accuracy of the crystal structure the statistical quantities R_free_ and R_work_ were used [46]. For occupancy refinement the occupancy of Val at P1 position was set to 0.5 and 3 rounds of occupancy refinement in Phenix were performed. The coordinates and structure factors have been deposited in the Protein Data Bank (entry code 4I5B). Figures depicting protein structures are generated with PyMOL (Schrodinger).

### Surface Plasmon Resonance

SPR experiments were conducted on a BIAcore 3000 machine (GE Healthcare) using streptavidin chips (GE Healthcare), which were primed and normalized before immobilization of biotinylated protein. For immobilization biotinylated wild-type DM and mutant DM (αR98A, αR194A; DM mut) were separately injected in two consecutive flow cells after diluting the protein to a concentration of 0.5 mg/mL in HBS-EP buffer (GE Healthcare). 500 RU (response units) of protein were immobilized in each flow cell using a flow rate of 10 µL/min at 25 ^°^C. Experiments were carried out in degassed 50 mM citrate phosphate buffer (pH 5.3), 150 mM NaCl and 0.06% C_12_E_9_ detergent (Calbiochem; EMD Chemicals). DR:peptide complexes (analyte) were diluted into the running buffer before injection and injected at 2 µM or the indicated concentration followed by buffer (15 µL/min, 25 ^°^C). Chips were regenerated by injection of pHA^P-2-P11^ (50 µM). Binding of the reference flow cell (DM mut) was subtracted from binding in the flow cell of DM wt.

### Peptide binding assay

For FP experiments cleaved DR1:CLIP_low_ was incubated with pHA^P-2-P11 [P5Alexa]^ labeled at position P5 (lysine-to-cysteine substitution) with a maleimide derivative of Alexa-488 (Molecular Probes). Excess peptide was separated by gel filtration using Bio-Spin 30 Tris columns (Bio-Rad Laboratories). FP experiments were conducted on a Victor^3^ V multilabel plate reader (PerkinElmer) using black polystyrene 384-well flat-bottomed plates (Corning). DR1:pHA^P-2-P11 [P5Alexa]^ complexes (100 nM) were incubated with a molar excess (50 µM) of unlabeled pHA^P-2-P11^ in the absence and presence of DM (100 nM). Measurements were performed in triplicates in a volume of 40 µL in 50 mM citrate phosphate buffer (pH 5.3) containing 150 mM NaCl at 25 ^°^C.

### Mass Spectrometry

To release covalently linked peptide from DR1 (20 µg) for analysis by mass spectrometry, the complex was incubated for 30 minutes at 25 ^°^C in the presence of 4 mM dithiothreitol. After addition of 20-fold molar excess of unlabeled pHA^P-2-P11^, the complex was incubated for an additional hour. Free DNP-labeled peptide was separated by anti-DNP chromatography and lyophilized. To analyze the peptide bound to DR1 in the crystal, DTT (40 mM) was added to isolated crystals and incubated over night at 25 ^°^C. Samples were submitted to the Dana-Farber Cancer Institute Molecular Biology Core Facility for MALDI-TOF analysis and liquid chromatography-tandem mass spectroscopy analysis.

## References

[1] Kaiko GE, Horvat JC, Beagley KW, Hansbro PM (2008) Immunological decision-making: how does the immune system decide to mount a helper T-cell response? Immunology 123: 326-338. doi:10.1111/j.1365-2567.2007.02719.x. PubMed: 17983439. 17983439 10.1111/j.1365-2567.2007.02719.x PMC2433332

[2] Mempel TR, Henrickson SE, Von Andrian UH (2004) T-cell priming by dendritic cells in lymph nodes occurs in three distinct phases. Nature 427: 154-159. doi:10.1038/nature02238. PubMed: 14712275. 14712275 10.1038/nature02238

[3] Lanzavecchia A, Reid PA, Watts C (1992) Irreversible association of peptides with class II MHC molecules in living cells. Nature 357: 249-252. doi:10.1038/357249a0. PubMed: 1375347. 1375347 10.1038/357249a0

[4] Roche PA, Cresswell P (1990) High-affinity binding of an influenza hemagglutinin-derived peptide to purified HLA-DR. J Immunol 144: 1849-1856. PubMed: 2307844. 2307844

[5] Rabinowitz JD, Vrljic M, Kasson PM, Liang MN, Busch R et al. (1998) Formation of a highly peptide-receptive state of class II MHC. Immunity 9: 699-709. doi:10.1016/S1074-7613(00)80667-6. PubMed: 9846491. 9846491 10.1016/s1074-7613(00)80667-6

[6] Germain RN, Rinker AG Jr. (1993) Peptide binding inhibits protein aggregation of invariant-chain free class II dimers and promotes surface expression of occupied molecules. Nature 363: 725-728. doi:10.1038/363725a0. PubMed: 8515815. 8515815 10.1038/363725a0

[7] Morris P, Shaman J, Attaya M, Amaya M, Goodman S et al. (1994) An essential role for HLA-DM in antigen presentation by class II major histocompatibility molecules. Nature 368: 551-554. doi:10.1038/368551a0. PubMed: 8139689. 8139689 10.1038/368551a0

[8] Roche PA, Cresswell P (1990) Invariant chain association with HLA-DR molecules inhibits immunogenic peptide binding. Nature 345: 615-618. doi:10.1038/345615a0. PubMed: 2190094. 2190094 10.1038/345615a0

[9] Avva RR, Cresswell P (1994) In vivo and in vitro formation and dissociation of HLA-DR complexes with invariant chain-derived peptides. Immunity 1: 763-774. doi:10.1016/S1074-7613(94)80018-9. PubMed: 7895165. 7895165 10.1016/s1074-7613(94)80018-9

[10] Denzin LK, Cresswell P (1995) HLA-DM induces CLIP dissociation from MHC class II alpha beta dimers and facilitates peptide loading. Cell 82: 155-165. doi:10.1016/0092-8674(95)90061-6. PubMed: 7606781. 7606781 10.1016/0092-8674(95)90061-6

[11] Sloan VS, Cameron P, Porter G, Gammon M, Amaya M et al. (1995) Mediation by HLA-DM of dissociation of peptides from HLA-DR. Nature 375: 802-806. doi:10.1038/375802a0. PubMed: 7596415. 7596415 10.1038/375802a0

[12] Sherman MA, Weber DA, Jensen PE (1995) DM enhances peptide binding to class II MHC by release of invariant chain-derived peptide. Immunity 3: 197-205. doi:10.1016/1074-7613(95)90089-6. PubMed: 7648393. 7648393 10.1016/1074-7613(95)90089-6

[13] Belmares MP, Busch R, Wucherpfennig KW, McConnell HM, Mellins ED (2002) Structural factors contributing to DM susceptibility of MHC class II/peptide complexes. J Immunol 169: 5109-5117. PubMed: 12391227. 12391227 10.4049/jimmunol.169.9.5109

[14] Weber DA, Evavold BD, Jensen PE (1996) Enhanced dissociation of HLA-DR-bound peptides in the presence of HLA-DM. Science 274: 618-620. doi:10.1126/science.274.5287.618. PubMed: 8849454. 8849454 10.1126/science.274.5287.618

[15] Vogt AB, Kropshofer H, Moldenhauer G, Hämmerling GJ (1996) Kinetic analysis of peptide loading onto HLA-DR molecules mediated by HLA-DM. Proc Natl Acad Sci U S A 93: 9724-9729. doi:10.1073/pnas.93.18.9724. PubMed: 8790398. 8790398 10.1073/pnas.93.18.9724 PMC38496

[16] Zarutskie JA, Busch R, Zavala-Ruiz Z, Rushe M, Mellins ED et al. (2001) The kinetic basis of peptide exchange catalysis by HLA-DM. Proc Natl Acad Sci U S A 98: 12450-12455. doi:10.1073/pnas.211439398. PubMed: 11606721. 11606721 10.1073/pnas.211439398 PMC60074

[17] Pos W, Sethi DK, Call MJ, Schulze MS, Anders AK et al. (2012) Crystal Structure of the HLA-DM-HLA-DR1 Complex Defines Mechanisms for Rapid Peptide Selection. Cell 151: 1557-1568. doi:10.1016/j.cell.2012.11.025. PubMed: 23260142. 23260142 10.1016/j.cell.2012.11.025 PMC3530167

[18] Chou CL, Sadegh-Nasseri S (2000) HLA-DM recognizes the flexible conformation of major histocompatibility complex class II. J Exp Med 192: 1697-1706. doi:10.1084/jem.192.12.1697. PubMed: 11120767. 11120767 10.1084/jem.192.12.1697 PMC2213500

[19] Anders AK, Call MJ, Schulze MS, Fowler KD, Schubert DA et al. (2011) HLA-DM captures partially empty HLA-DR molecules for catalyzed removal of peptide. Nat Immunol 12: 54-61. doi:10.1038/ni.1967. PubMed: 21131964. 21131964 10.1038/ni.1967 PMC3018327

[20] Sadegh-Nasseri S, Germain RN (1991) A role for peptide in determining MHC class II structure. Nature 353: 167-170. doi:10.1038/353167a0. PubMed: 1653903. 1653903 10.1038/353167a0

[21] Natarajan SK, Stern LJ, Sadegh-Nasseri S (1999) Sodium dodecyl sulfate stability of HLA-DR1 complexes correlates with burial of hydrophobic residues in pocket 1. J Immunol 162: 3463-3470. PubMed: 10092802. 10092802

[22] Sato AK, Zarutskie JA, Rushe MM, Lomakin A, Natarajan SK et al. (2000) Determinants of the peptide-induced conformational change in the human class II major histocompatibility complex protein HLA-DR1. J Biol Chem 275: 2165-2173. doi:10.1074/jbc.275.3.2165. PubMed: 10636922. 10636922 10.1074/jbc.275.3.2165

[23] Jardetzky TS, Brown JH, Gorga JC, Stern LJ, Urban RG et al. (1996) Crystallographic analysis of endogenous peptides associated with HLA-DR1 suggests a common, polyproline II-like conformation for bound peptides. Proc Natl Acad Sci U S A 93: 734-738. doi:10.1073/pnas.93.2.734. PubMed: 8570625. 8570625 10.1073/pnas.93.2.734 PMC40123

[24] Brown JH, Jardetzky TS, Gorga JC, Stern LJ, Urban RG et al. (1993) Three-dimensional structure of the human class II histocompatibility antigen HLA-DR1. Nature 364: 33-39. doi:10.1038/364033a0. PubMed: 8316295. 8316295 10.1038/364033a0

[25] Stratikos E, Wiley DC, Stern LJ (2004) Enhanced catalytic action of HLA-DM on the exchange of peptides lacking backbone hydrogen bonds between their N-terminal region and the MHC class II alpha-chain. J Immunol 172: 1109-1117. PubMed: 14707085. 14707085 10.4049/jimmunol.172.2.1109

[26] Narayan K, Chou CL, Kim A, Hartman IZ, Dalai S et al. (2007) HLA-DM targets the hydrogen bond between the histidine at position beta81 and peptide to dissociate HLA-DR-peptide complexes. Nat Immunol 8: 92-100. doi:10.1038/nrg2061. PubMed: 17143275. 17143275 10.1038/ni1414 PMC3019572

[27] Zhou Z, Callaway KA, Weber DA, Jensen PE (2009) Cutting edge: HLA-DM functions through a mechanism that does not require specific conserved hydrogen bonds in class II MHC-peptide complexes. J Immunol 183: 4187-4191. doi:10.4049/jimmunol.0901663. PubMed: 19767569. 19767569 10.4049/jimmunol.0901663 PMC2912111

[28] Ferrante A, Gorski. (2010) J Cutting edge: HLA-DM-mediated peptide exchange functions normally on MHC class II-peptide complexes that have been weakened by elimination of a conserved hydrogen bond. J Immunol 184: 1153-1158. doi:10.4049/jimmunol.0902878. PubMed: 20038641. 20038641 10.4049/jimmunol.0902878

[29] Stern LJ, Brown JH, Jardetzky TS, Gorga JC, Urban RG et al. (1994) Crystal structure of the human class II MHC protein HLA-DR1 complexed with an influenza virus peptide. Nature 368: 215-221. doi:10.1038/368215a0. PubMed: 8145819. 8145819 10.1038/368215a0

[30] Ullrich HJ, Döring K, Grüneberg U, Jähnig F, Trowsdale J et al. (1997) Interaction between HLA-DM and HLA-DR involves regions that undergo conformational changes at lysosomal pH. Proc Natl Acad Sci U S A 94: 13163-13168. doi:10.1073/pnas.94.24.13163. PubMed: 9371817. 9371817 10.1073/pnas.94.24.13163 PMC24280

[31] Adams PD, Afonine PV, Bunkóczi G, Chen VB, Davis IW et al. (2010) PHENIX: a comprehensive Python-based system for macromolecular structure solution. Acta Crystallogr D Biol Crystallogr 66: 213-221. doi:10.1107/S0907444909052925. PubMed: 20124702. 20124702 10.1107/S0907444909052925 PMC2815670

[32] Nicholson MJ, Moradi B, Seth NP, Xing X, Cuny GD et al. (2006) Small molecules that enhance the catalytic efficiency of HLA-DM. J Immunol 176: 4208-4220. PubMed: 16547258. 16547258 10.4049/jimmunol.176.7.4208 PMC3412064

[33] McFarland BJ, Katz JF, Beeson C, Sant AJ (2001) Energetic asymmetry among hydrogen bonds in MHC class II*peptide complexes. Proc Natl Acad Sci U S A 98: 9231-9236. doi:10.1073/pnas.151131498. PubMed: 11470892. 11470892 10.1073/pnas.151131498 PMC55403

[34] Painter CA, Negroni MP, Kellersberger KA, Zavala-Ruiz Z, Evans JE et al. (2011) Conformational lability in the class II MHC 310 helix and adjacent extended strand dictate HLA-DM susceptibility and peptide exchange. Proc Natl Acad Sci U S A 108: 19329-19334. doi:10.1073/pnas.1108074108. PubMed: 22084083. 22084083 10.1073/pnas.1108074108 PMC3228433

[35] Hammer J, Belunis C, Bolin D, Papadopoulos J, Walsky R et al. (1994) High-affinity binding of short peptides to major histocompatibility complex class II molecules by anchor combinations. Proc Natl Acad Sci U S A 91: 4456-4460. doi:10.1073/pnas.91.10.4456. PubMed: 8183931. 8183931 10.1073/pnas.91.10.4456 PMC43804

[36] Doebele RC, Busch R, Scott HM, Pashine A, Mellins ED (2000) Determination of the HLA-DM interaction site on HLA-DR molecules. Immunity 13: 517-527. doi:10.1016/S1074-7613(00)00051-0. PubMed: 11070170. 11070170 10.1016/s1074-7613(00)00051-0

[37] Pashine A, Busch R, Belmares MP, Munning JN, Doebele RC et al. (2003) Interaction of HLA-DR with an acidic face of HLA-DM disrupts sequence-dependent interactions with peptides. Immunity 19: 183-192. doi:10.1016/S1074-7613(03)00200-0. PubMed: 12932352. 12932352 10.1016/s1074-7613(03)00200-0

[38] Stratikos E, Mosyak L, Zaller DM, Wiley DC (2002) Identification of the lateral interaction surfaces of human histocompatibility leukocyte antigen (HLA)-DM with HLA-DR1 by formation of tethered complexes that present enhanced HLA-DM catalysis. J Exp Med 196: 173-183. doi:10.1084/jem.20020117. PubMed: 12119342. 12119342 10.1084/jem.20020117 PMC2193930

[39] Denzin LK, Hammond C, Cresswell P (1996) HLA-DM interactions with intermediates in HLA-DR maturation and a role for HLA-DM in stabilizing empty HLA-DR molecules. J Exp Med 184: 2153-2165. doi:10.1084/jem.184.6.2153. PubMed: 8976171. 8976171 10.1084/jem.184.6.2153 PMC2196380

[40] Kropshofer H, Vogt AB, Moldenhauer G, Hammer J, Blum JS et al. (1996) Editing of the HLA-DR-peptide repertoire by HLA-DM. EMBO J 15: 6144-6154. PubMed: 8947036. 8947036 PMC452435

[41] van Ham SM, Grüneberg U, Malcherek G, Bröker I, Melms A et al. (1996) Human histocompatibility leukocyte antigen (HLA)-DM edits peptides presented by HLA-DR according to their ligand binding motifs. J Exp Med 184: 2019-2024. doi:10.1084/jem.184.5.2019. PubMed: 8920889. 8920889 10.1084/jem.184.5.2019 PMC2192865

[42] Otwinowski Z, Minor W (1997) Processing of X-ray diffraction data collected in oscillation mode. Macromol Crystallogr Pt A 276: 307-326. doi:10.1016/S0076-6879(97)76066-X. 10.1016/S0076-6879(97)76066-X 27754618

[43] Mccoy AJ, Grosse-Kunstleve RW, Adams PD, Winn MD, Storoni LC et al. (2007) Phaser crystallographic software. J Appl Crystallogr 40: 658-674. doi:10.1107/S0021889807021206. PubMed: 19461840. 19461840 10.1107/S0021889807021206 PMC2483472

[44] Chen VB, Arendall WB, Headd JJ, Keedy DA, Immormino RM et al. (2010) MolProbity: all-atom structure validation for macromolecular crystallography. Acta Crystallogr D Biol Crystallogr 66: 12-21. doi:10.1107/S1744309109042018. PubMed: 20057044. 20057044 10.1107/S0907444909042073 PMC2803126

[45] Emsley P, Cowtan K (2004) Coot: model-building tools for molecular graphics. Acta Crystallogr D Biol Crystallogr 60: 2126-2132. doi:10.1107/S0907444904019158. PubMed: 15572765. 15572765 10.1107/S0907444904019158

[46] Brünger AT (1992) Free R-Value - a Novel Statistical Quantity for Assessing the Accuracy of Crystal-Structures. Nature 355: 472-475. doi:10.1038/355472a0. PubMed: 18481394. 18481394 10.1038/355472a0

